# Hole interactions of aerogen oxides with Lewis bases: an insight into σ-hole and lone-pair-hole interactions

**DOI:** 10.1098/rsos.231362

**Published:** 2023-12-13

**Authors:** Mahmoud A. A. Ibrahim, Mohammed N. I. Shehata, Hassan A. A. Abuelliel, Nayra A. M. Moussa, Shaban R. M. Sayed, Muhammad Naeem Ahmed, Mohamed K. Abd El-Rahman, Eslam Dabbish, Tamer Shoeib

**Affiliations:** ^1^ Computational Chemistry Laboratory, Chemistry Department, Faculty of Science, Minia University, Minia 61519, Egypt; ^2^ School of Health Sciences, University of KwaZulu-Natal, Westville Campus, Durban 4000, South Africa; ^3^ Department of Botany and Microbiology, College of Science, King Saud University, PO Box 2455, Riyadh 11451, Saudi Arabia; ^4^ Department of Chemistry, The University of Azad Jammu and Kashmir, Muzaffarabad 13100, Pakistan; ^5^ Department of Chemistry and Chemical Biology, Harvard University, 12 Oxford Street, Cambridge, MA 02138, USA; ^6^ Department of Chemistry, The American University in Cairo, New Cairo 11835, Egypt

**Keywords:** σ-hole interactions, lone-pair-hole interactions, symmetry-adapted perturbation theory analysis, point-of-charge analysis, aerogen oxides

## Abstract

σ-Hole and lone-pair (lp)-hole interactions of aerogen oxides with Lewis bases (LB) were comparatively inspected in terms of quantum mechanics calculations. The ZO_n_ ⋯ LB complexes (where Z = Kr and Xe, *n* = 1, 2, 3 and 4, and LB = NH_3_ and NCH) showed favourable negative interaction energies. The complexation features were explained in light of σ-hole and lp-hole interactions within optimum distances lower than the sum of the respective van der Waals radii. The emerging findings outlined that σ-hole interaction energies generally enhanced according to the following order: KrO_4_ ⋯ < KrO⋯ < KrO_3_⋯ < KrO_2_⋯LB and XeO_4_⋯ < XeO⋯ < XeO_2_⋯ < XeO_3_⋯LB complexes with values ranging from –2.23 to –12.84 kcal mol^−1^. Lp-hole interactions with values up to –5.91 kcal mol^−1^ were shown. Symmetry-adapted perturbation theory findings revealed the significant contributions of electrostatic forces accounting for 50–65% of the total attractive forces within most of the ZO_n_⋯LB complexes. The obtained observations would be useful for the understanding of hole interactions, particularly for the aerogen oxides, with application in supramolecular chemistry and crystal engineering.

## 1. Introduction

Noncovalent interactions received significant attention in fields such as supramolecular chemistry [1,2], drug discovery [3], self-assembly [4–6] and crystal engineering [7]. Noncovalent interaction strength extends from the weak van der Waals forces to ionic bonds [8]. Among noncovalent interactions, hole interactions of most recent interest were ubiquitous and of eminent importance. Basically, hole interactions are categorized based on the origin of the covalent orbital as σ-hole [9,10], π-hole [11,12], lone-pair (lp)-hole [13,14], or R^•^-hole [15]. As a member of the hole interactions family, the σ-hole concept was first announced to describe an electron-deficient region that existed along the extension of the covalently bonded group VII atoms [16–19]. Such concept was then extended to Group IV–VIII elements, forming tetrels [20–24], pnicogens [25–28], chalcogens [29–32], halogens [33–39] and aerogens [40,41] bonding complexes.

More recently, significant efforts have been exerted in investigating unconventional hole interactions with aerogen (i.e. noble gas)-bearing compounds owing to their crucial role in supramolecular chemistry and crystal engineering [42]. It is worth mentioning that the noncovalent interactions involving xenon oxides and fluorides were documented with a significant role in directing the self-assembly of supramolecular structures and the crystallization of new materials [43]. The chemistry of noble gases was initiated in 1962 when XeF_2_ and XePtF_6_ compounds were discovered [44]. The synthesis of [AuXe_4_]^2+^ and [XeAuF] compounds in 2000 [45] and 2004 [46], respectively, opened the door to further exploration of noble gas interactions [47,48].

By 2015, Bauzá and Frontera pointed to the ability of Group VIII elements to engage in hole interactions and hence form what was termed aerogen bonds [40,49] supported by solid-state structures [50,51] as well as experimental and theoretical studies to identify the characteristics of aerogen bonding [51–58]. Principally, a set of X-ray structures characterizing the σ-hole interactions versus the π-hole analogues of xenon fluorides XeF*_n_* (where *n* = 2, 3, 4, 5, and 6) were rigorously deliberated via the Cambridge structural database (CSD) [50]. In this regard, the energetic features demonstrated the preferentiality of the XeF*_n_* ⋯ Lewis base interactions in the order XeF_2_ ⋯ < XeF_4_ ⋯ < XeF_6_ ⋯ < XeF_5_^+^ ⋯ < XeF_3_^+^ ⋯ Lewis base complexes. Previous work was limited to aerogen trioxide (ZO_3_, where Z = Kr or Xe) through their bonding sites, namely, σ-hole [41,59–61] and lp-hole [61] sites. In a comparative study, the potentiality of the aerogen-bearing molecules to favourably interact via their σ-hole and π-hole sites with C_2_H_4_ Lewis base was adequately addressed with further preferability demonstrated for the σ-hole based interactions [62].

**Figure 1.**
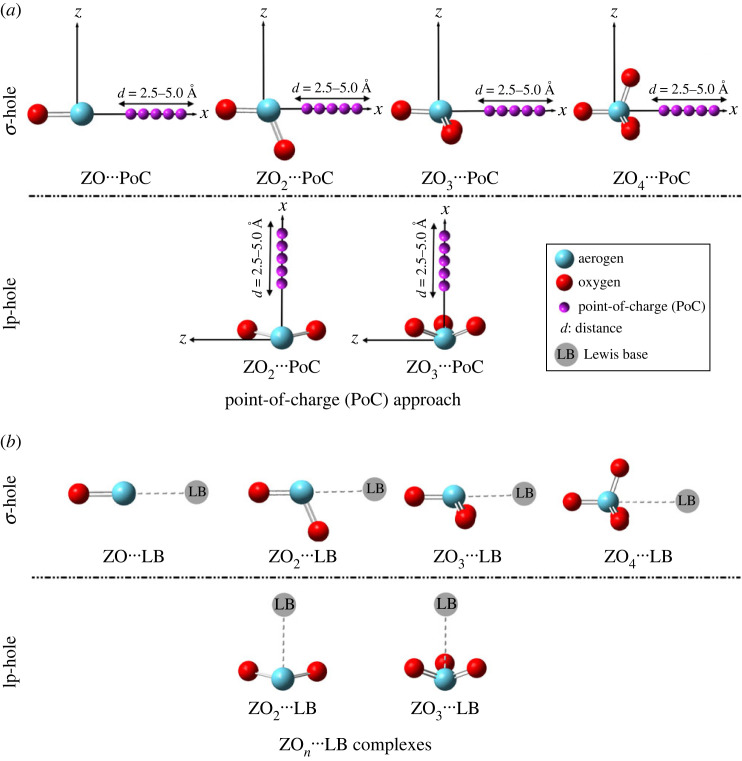
Schematic modelling of (*a*) the point-of-charge (PoC) calculations for the ZO*_n_* ⋯ PoC systems and (*b*) the ZO*_n_* ⋯ LB complexes (where Z = Kr and Xe, *n* = 1, 2, 3 and 4, and LB = NH_3_ and NCH).

The present study was accordingly dedicated to investigating the prospected hole interactions within ZO*_n_* ⋯ LB complexes (where Z = Kr and Xe, *n* = 1, 2, 3 and 4, and LB = NH_3_ and NCH). Chiefly, geometrical optimization was performed for the ZO ⋯ LB (σ-hole), ZO_2_ ⋯ LB (σ-hole/lp-hole), ZO_3_ ⋯ LB (σ-hole/lp-hole), and ZO_4_ ⋯ LB (σ-hole) complexes (figure 1) accompanied by a set of energetic calculations.

The studied interactions were quantitatively and qualitatively identified through the quantum theory of atoms in molecules (QTAIM) and noncovalent interaction (NCI) index analyses. The prevalent forces beyond the inspected interactions were discussed using the symmetry-adapted perturbation theory (SAPT) analysis. The findings manifested a supreme ground for future investigations related to the hole interactions of aerogen oxides with Lewis bases.

## 2. Results and discussion

### 2.1. Electrostatic potential analyses

**Figure 2.**
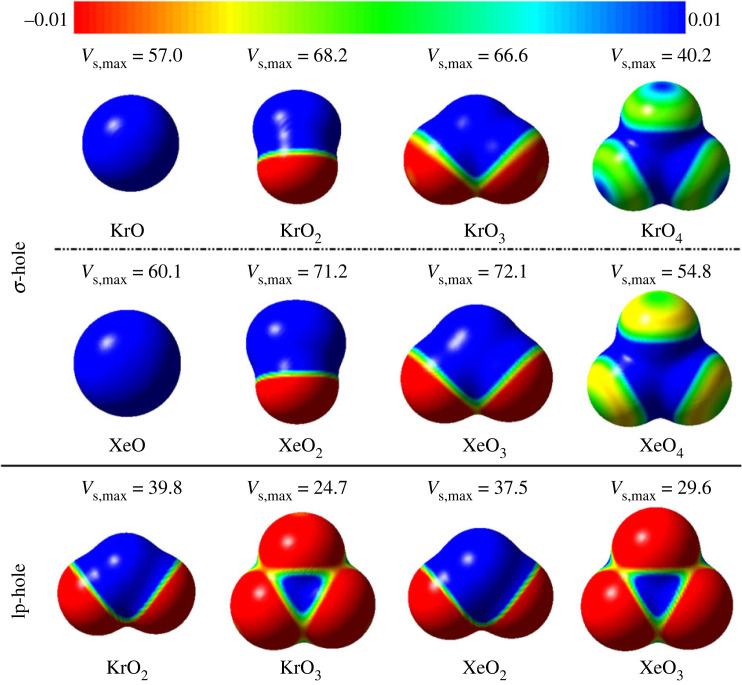
Charge distribution over the entities of the aerogen oxides (ZO*_n_*, where Z = Kr and Xe, and *n* = 1, 2, 3, and 4) in the MEP maps. The EP aligned in the +0.01 au (blue) and –0.01 au (red) ambit. The *V*_s,max_ values are in kcal mol^−1^.

Electrostatic potential (EP) analysis was demonstrated to be able to identify the charge distribution locations of the electrophilic and nucleophilic regions in molecular systems [63,64]. Figure 2 demonstrates the size and magnitude of the σ-hole and lp-hole of the ZO*_n_* molecules in the fashion of molecular electrostatic potential (MEP) maps and the corresponding maximum positive electrostatic potential (*V*_s,max_) values.

As given in figure 2, blue-coded areas were found over the aerogen atoms with disparate sizes that varied from one geometrical structure to another, reflecting the existence of different holes. Principally, σ-holes were detected along the outer surface of the O–Z covalent bonds of the modelled linear (ZO), bent (ZO_2_), trigonal pyramidal (ZO_3_), and tetrahedral structures (ZO_4_). While lp-hole was only apparent on the extension of aerogen lone pair of electrons within the bent and trigonal pyramidal structures of the ZO_2_ and ZO_3_ molecules, respectively. Overall, the tendency of the ZO*_n_* systems to engage in σ-hole interactions and, in particular, the ZO_2_/ZO_3_ molecules in lp-hole interactions was primarily confirmed.

EP analyses of σ-hole bearing molecules showed larger σ-hole sizes for the XeO*_n_* molecules relative to those of KrO*_n_*. For instance, the *V*_s,max_ of KrO_2_ and XeO_2_ molecules were 68.2 and 71.2 kcal mol^−1^, respectively. Regarding KrO*_n_* molecules, EP findings indicated the order of σ-hole size pattern according to the following pattern: bent < trigonal < pyramidal < linear < tetrahedral structures. The *V*_s,max_ values of KrO_2_, KrO_3_, KrO, and KrO_4_ were 68.2, 66.6, 57.0 and 40.2 kcal mol^−1^, respectively. While the size of the σ-holes of XeO*_n_* molecules decreased according to the following order: XeO_3_ (trigonal pyramidal) > XeO_2_ (bent) > XeO (linear) > XeO_4_ (tetrahedral) with magnitudes of 72.2, 71.1, 60.1 and 54.8 kcal mol^−1^, respectively.

For lp-hole-bearing molecules, larger lp-hole sizes were noted within the bent structures of ZO_2_ compared with the trigonal pyramidal one of ZO_3_, highlighting the more significant bias of the former molecule to participate in lp-hole interactions than the latter one. As an illustration, the *V*_s,max_ for the KrO_2_ and KrO_3_ molecules was determined to be 39.8 and 24.7 kcal mol^−1^, respectively. Overall, the ZO_2_ and ZO_3_ molecules showed an increasing ability to participate in σ-hole interactions relative to their lp-hole counterparts.

To provide an in-depth insight into the polarization effect on the studied aerogen-bearing molecules, MEP maps were generated for the investigated ZO*_n_* (where Z = Kr and Xe, and *n* = 1, 2, 3, and 4) monomers in the presence of negative PoC with a value of –0.75 au (electronic supplementary material, figure S1).

According to the displayed maps in the electronic supplementary material, figure S1, it is clear that the size of both studied holes in the presence of negative PoC showed higher positive blue-coded regions in comparison with their analogues in figure 2.

### 2.2. Point-of-charge analysis

**Figure 3.**
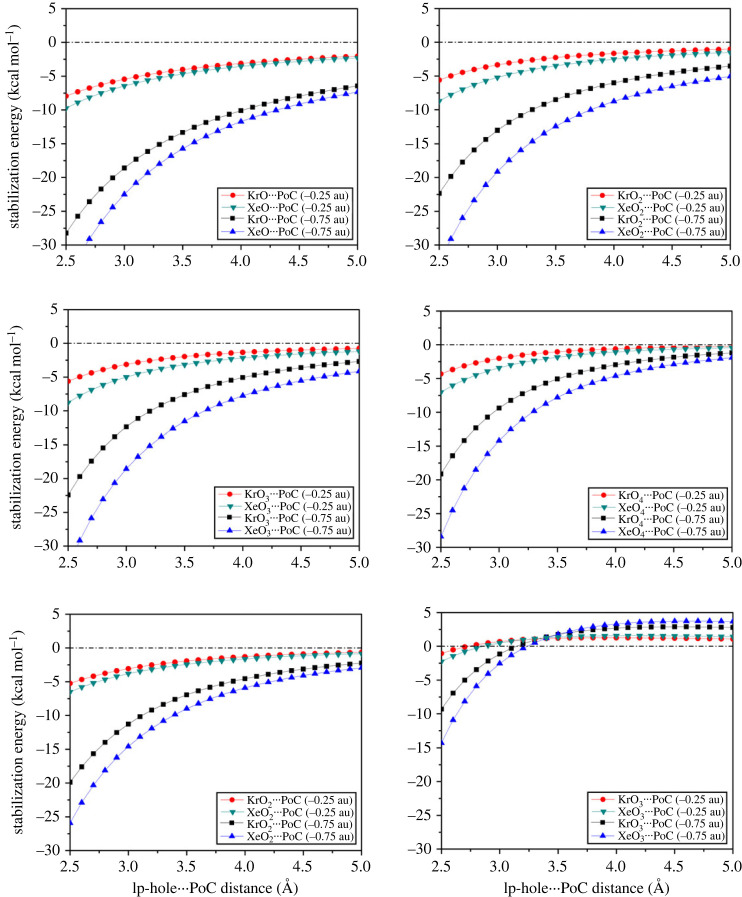
Stabilization energy curves of ZO*_n_* ⋯ PoC systems (where Z = Kr and Xe, and *n* = 1, 2, 3, and 4) under the effect of PoC = –0.25 and –0.75 au using σ-hole ⋯ /lp-hole ⋯ PoC distance ambit of 2.5–5.0 Å within 0.1 Å step size.

The point-of-charge (PoC) approach has recently been considered a valid approach to study the ability of hole-containing molecules, including σ- [65–67], π- [65], lp- [13], and R^•^-holes [15], to engage in noncovalent interactions from an electrostatic perspective. The PoC approach was used to explicitly account for the polarization effect on hole-containing molecules [68]. In the PoC context, stabilization energies (*E*_stabilization_) were assessed for the ZO*_n_* ⋯ PoC systems under the effect of PoC = –0.25 and –0.75 au using σ-hole ⋯ /lp-hole ⋯ PoC distance ambit of 2.5–5.0 Å within 0.1 Å step size (figure 3).

Figure 3 shows the potentiality of the studied aerogen-containing molecules to participate in σ-hole and lp-hole interactions from an electrostatic perspective. In particular, the σ-hole ⋯ PoC systems showed significant *E*_stabilization_ with higher negative values than that of the lp-hole. Besides, the *E*_stabilization_ was shown to attenuate with increasing the σ-hole/lp-hole ⋯ PoC distances. Notable growth in the *E*_stabilization_ was observed along with swelling the selected PoCs negativity and decreasing the electronegativity of the investigated aerogens (i.e. Xe > Kr). It is worth noting that the *E*_stabilization_ was found to follow the order of KrO > KrO_3_ > KrO_2_ > KrO_4_ and XeO > XeO_2_ > XeO_3_ > XeO_4_. Such orders are slightly different from those observed in the EP computations. This finding could be explained as an upshot of the inability of EP analysis to consider the polarization effect.

### 2.3. Energetic study

**Figure 4.**
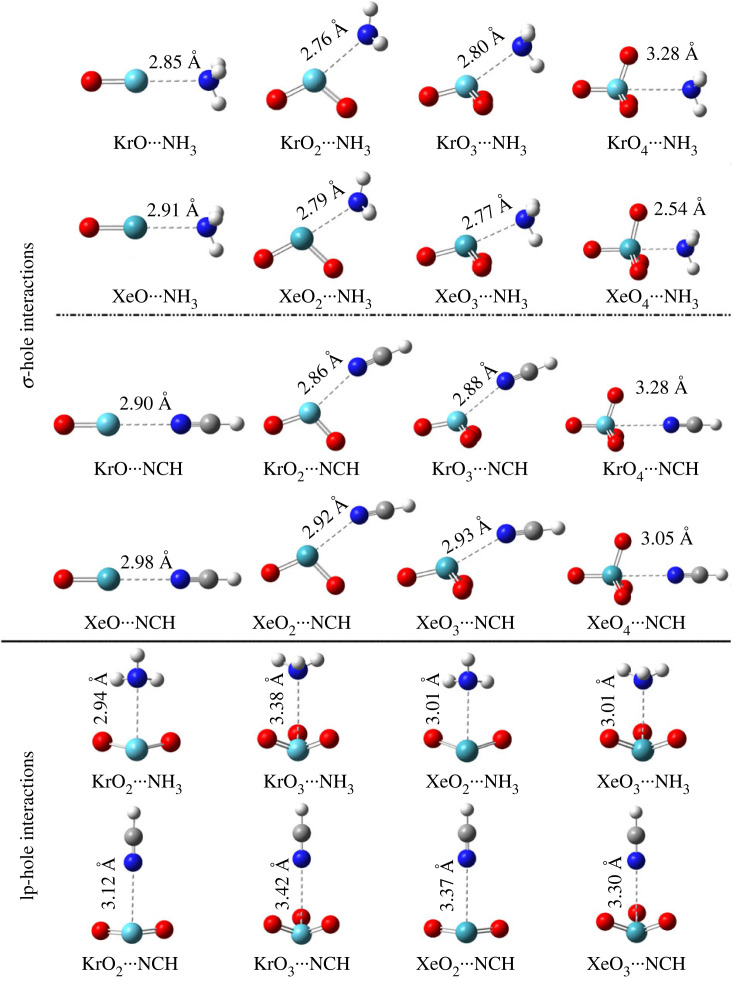
Optimized structures of ZO*_n_* ⋯ LB complexes (where Z = Kr and Xe, *n* = 1, 2, 3, and 4, and LB = NH_3_ and NCH). σ-hole/lp-hole ⋯ N distances are in Å.

**Figure 5.**
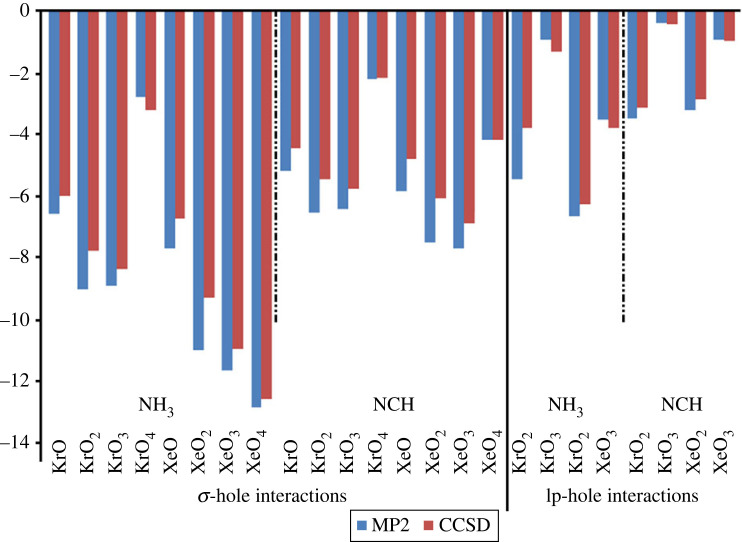
Bar chart showing the obtained *E*_int_ at the MP2/aug-cc-pVTZ(PP) level of theory versus the CCSD(T)/CBS analogue of ZO*_n_* ⋯ LB complexes (where Z = Kr and Xe, *n* = 1, 2, 3 and 4, and LB = NH_3_ and NCH).

σ-Hole and lp-hole interactions were studied within the ZO*_n_* ⋯ LB complexes (where Z = Kr and Xe, *n* = 1, 2, 3, and 4, and LB = NH_3_ and NCH). Applying the MP2/aug-cc-pVTZ(PP) computational level, geometrical optimization was first executed escorted by the calculations of interaction energy (*E*_int_). Figure 4 shows the ZO*_n_* ⋯ LB complexes and their corresponding optimum distances. The correlation between the MP2 energies and their CCSD(T)/CBS counterparts is graphically elucidated in figure 5. The calculated interaction energies (*E*_int_) are listed in table 1.

**Table 1.** *E*_int_ values of the ZO*_n_* ⋯ LB complexes (where Z = Kr and Xe, *n* = 1, 2, 3, and 4, and LB = NH_3_ and NCH) are evaluated in kcal mol^−1^ at the MP2/aug-cc-pVTZ(PP) level. The σ-hole/lp-hole ⋯ N distances are in Å.

complex	complexation parameters		complex	complexation parameters	
	distance (Å)	*E*_int_ (kcal mol^−1^)		distance (Å)	*E*_int_ (kcal mol^−1^)
σ-hole interactions					
KrO ⋯ NH_3_	2.85	–6.57	KrO ⋯ NCH	2.90	–5.17
KrO_2_ ⋯ NH_3_	2.76	–9.00	KrO_2_ ⋯ NCH	2.86	–6.52
KrO_3_ ⋯ NH_3_	2.80	–8.87	KrO_3_ ⋯ NCH	2.88	–6.42
KrO_4_ ⋯ NH_3_	3.28	–2.78	KrO_4_ ⋯ NCH	3.28	–2.23
XeO ⋯ NH_3_	2.91	–7.70	XeO ⋯ NCH	2.98	–5.83
XeO_2_ ⋯ NH_3_	2.79	–10.98	XeO_2_ ⋯ NCH	2.92	–7.50
XeO_3_ ⋯ NH_3_	2.77	–11.65	XeO_3_ ⋯ NCH	2.93	–7.67
XeO_4_ ⋯ NH_3_	2.54	–12.84	XeO_4_ ⋯ NCH	3.05	–4.18
lp-hole interactions					
KrO_2_ ⋯ NH_3_	2.94	–5.91	KrO_2_ ⋯ NCH	3.12^a^	–3.48^a^
KrO_3_ ⋯ NH_3_	3.38	–0.92	KrO_3_ ⋯ NCH	3.42	–0.36
XeO_2_ ⋯ NH_3_	3.01	–6.63	XeO_2_ ⋯ NCH	3.37^a^	–3.20^a^
XeO_3_ ⋯ NH_3_	3.01	–3.50	XeO_3_ ⋯ NCH	3.30	–0.93

^a^*E*_int_ was computed from the potential energy surface (PES) scan.

According to the displayed data in figure 4, the studied aerogen-bearing molecules showed conspicuous potentiality to form σ- and lp-hole interactions with LBs on the extension of O–Z covalent bond and aerogen lone pair of electrons, respectively.

In general, each modelled structure of the KrO_*n*_ ⋯ and XeO_*n*_ ⋯ LB complexes showed a linear correlation between the energetic claims and the σ-hole size patterns, regardless of the XeO*_n_* ⋯ NH_3_ complexes. For instance, *E*_int_ of the KrO*_n_* ⋯ NH_3_ complexes were found to track those of the successive series KrO_2_ ⋯ > KrO_3_ ⋯ > KrO ⋯ > KrO_4_ ⋯ NH_3_, amounting to –9.00, –8.87, –6.57, and –2.78 kcal mol^−1^ versus *V_s_*_,max_ values of 68.2, 66.6, 57.0, and 40.2 kcal mol^−1^, respectively. The highest negative *E*_int_ along with the smallest σ-hole ⋯ N distance for the XeO*_n_* ⋯ NH_3_ complexes and the lowest *V*_s,max_ value was determined for *n* = 4. These results might be ascribed to the σ-hole ⋯ NH_3_ attractive forces over the repulsive ones of the coplanar oxygen atoms in XeO_4_ ⋯ NH_3_. Interaction energies of σ-hole interactions were shown to augment the atomic size of the inspected aerogens in the ZO*_n_* ⋯ LB complexes (table 1).

*E*_int_ were determined to be –5.17/–5.83, –6.52/–7.50, –6.42/–7.67 and –2.23/–4.18 kcal mol^−1^ for the KrO/XeO ⋯ , KrO_2_/XeO_2_ ⋯ , KrO_3_/XeO_3_ ⋯ , and KrO_4_/XeO_4_ ⋯ NCH complexes, respectively. Moreover, elevated negative values of *E*_int_ were detected for the ZO*_n_* ⋯ NH_3_ complexes in comparison to the ZO*_n_* ⋯ NCH counterparts, indicating the increased *E*_int_ with increased negativity of the Lewis bases. Illustratively, MP2 energies were –7.70/–5.83, –10.98/–7.50, –11.65/–7.67 and –12.84/–4.18 kcal mol^−1^ for the XeO ⋯ , XeO_2_ ⋯ , XeO_3_ ⋯ and XeO_4_ ⋯ NH_3_/NCH complexes, respectively. For the *E*_int_ results of the lp-hole interactions, the ZO_2_ molecules indicated their preferential lp-hole interactions over the ZO_3_ counterparts. The *E*_int_ values were determined for the KrO_2_ ⋯ and KrO_3_ ⋯ NCH complexes to be –3.48 and –0.36 kcal mol^−1^ along with *V*_s,max_ of 39.8 and 24.7 kcal mol^−1^ for the KrO_2_ and KrO_3_ molecules, respectively.

Comparing σ-hole and lp-hole energetic quantities of the ZO_2_ ⋯ and ZO_3_ ⋯ LB complexes showed significant *E*_int_ were recorded for σ-hole interactions relative to the lp-hole ones, aligning with their corresponding σ-hole and lp-hole magnitudes. As evidence, for the aforementioned interactions within the XO_3_ ⋯ NH_3_ complexes, the *E*_int_ were –11.65 and –3.50 kcal mol^−1^, along with σ-hole size of 72.1 kcal mol^−1^ and lp-hole one of 29.6 kcal mol^−1^, respectively.

Overall, all the examined ZO*_n_* molecules exhibited a preference to engage in interactions via their bonding sites within optimum distances that are lower than the sum of the corresponding van der Waals radii (figure 4). Inspecting the bar charts presented in figure 5 revealed a relative approximation between the CCSD(T)/CBS energetic claims and the MP2 candidates.

### 2.4. Quantum theory of atoms in molecules analysis

**Figure 6.**
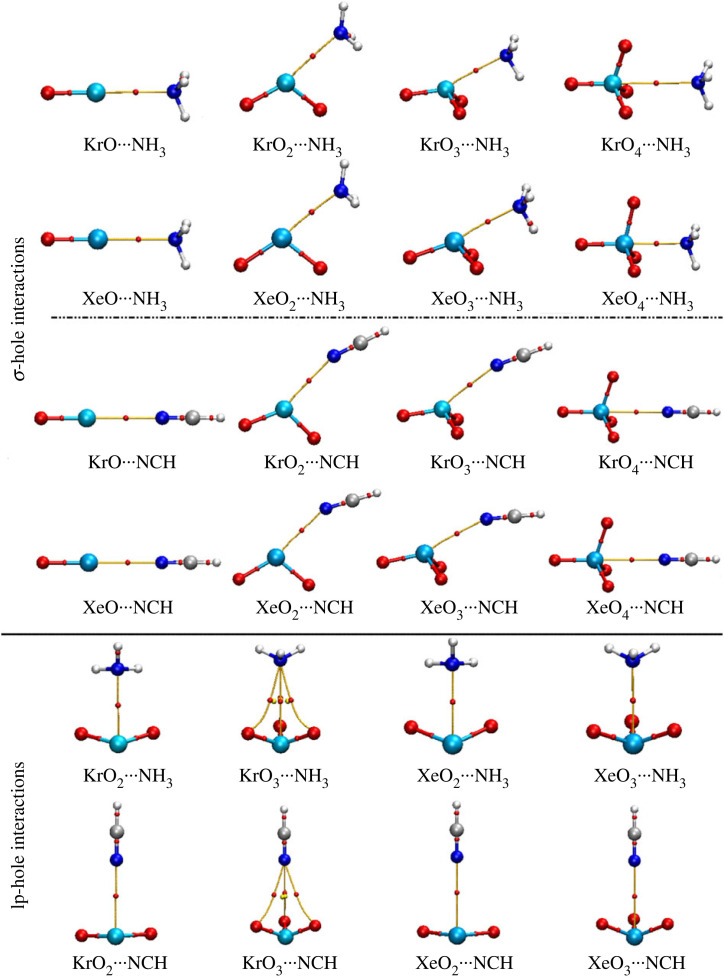
QTAIM schemes of the ZO*_n_* ⋯ LB complexes (where Z = Kr and Xe, *n* = 1, 2, 3 and 4, and LB = NH_3_ and NCH). BCP locations are indicated by red dots.

QTAIM was deemed a reliable approach to investigate the electron density features of the noncovalent interactions [69,70]. QTAIM schemes of the ZO*_n_* ⋯ LB complexes (where Z = Kr and Xe, *n* = 1, 2, 3 and 4, and LB = NH_3_ and NCH) are shown in figure 6. Topological parameters, comprising the *ρ*_b_, ∇^2^*ρ*_b_, and H_b_, are summarized in table 2.

**Table 2.** *ρ*_b_, ∇^2^*ρ*_b_, and H_b_ (in au) of the ZO*_n_* ⋯ LB complexes (where Z = Kr and Xe, *n* = 1, 2, 3, and 4, and LB = NH_3_ and NCH).

complex	*ρ*_b_ (au)	∇^2^*ρ*_b_ (au)	H_b_ (au)	complex	*ρ*_b_ (au)	∇^2^*ρ*_b_ (au)	H_b_ (au)
σ-hole interactions							
KrO ⋯ NH_3_	0.0205	0.0704	0.0015	KrO ⋯ NCH	0.0156	0.0639	0.0023
KrO_2_ ⋯ NH_3_	0.0243	0.0811	0.0010	KrO_2_ ⋯ NCH	0.0173	0.0685	0.0022
KrO_3_ ⋯ NH_3_	0.0236	0.0758	0.0009	KrO_3_ ⋯ NCH	0.0175	0.0670	0.0020
KrO_4_ ⋯ NH_3_	0.0092	0.0336	0.0012	KrO_4_ ⋯ NCH	0.0076	0.0328	0.0016
XeO ⋯ NH_3_	0.0235	0.0704	0.0005	XeO ⋯ NCH	0.0173	0.0638	0.0018
XeO_2_ ⋯ NH_3_	0.0294	0.0812	–0.0008	XeO_2_ ⋯ NCH	0.0196	0.0686	0.0016
XeO_3_ ⋯ NH_3_	0.0317	0.0801	–0.0015	XeO_3_ ⋯ NCH	0.0202	0.0673	0.0015
XeO_4_ ⋯ NH_3_	0.0529	0.0790	–0.0114	XeO_4_ ⋯ NCH	0.0149	0.0534	0.0017
lp-hole interactions							
KrO_2_ ⋯ NH_3_	0.0164	0.0599	0.0016	KrO_2_ ⋯ NCH	0.0112^a^	0.0438^a^	0.0019^a^
KrO_3_ ⋯ NH_3_	0.0074	0.0290	0.0012	KrO_3_ ⋯ NCH	0.0058	0.0218	0.0011
XeO_2_ ⋯ NH_3_	0.0185	0.0571	0.0010	XeO_2_ ⋯ NCH	0.0096^a^	0.0326^a^	0.0015^a^
XeO_3_ ⋯ NH_3_	0.0191	0.0560	0.0008	XeO_3_ ⋯ NCH	0.0089	0.0352	0.0017

^a^PES scan was considered for obtaining the QTAIM parameters at the most favourable parameters.

Looking at figure 6, the occurrence of the investigated σ-/lp-hole interactions within the ZO*_n_* ⋯ LB complexes was evinced through the presence of one BP and BCP between the aerogens at the extension of O–Z covalent bond/aerogen lone pair of electrons and Lewis bases. The closed-shell nature of the interactions involved within the ZO*_n_* ⋯ LB complexes was also proved through *ρ*_b_ with low values accompanied by ∇^2^*ρ*_b_ and H_b_ values with a positive sign (table 2). Exceptionally, the σ-hole interactions of XeO_2_ ⋯ , XeO_3_ ⋯ , and XeO_4_ ⋯ NH_3_ complexes showed negative H_b_ values, confirming their partial covalent nature.

From table 2, the *ρ*_b_, ∇^2^*ρ*_b_, and H_b_ trends of the σ-hole interactions generally pinpointed a great consistency with the corresponding *E*_int_ features. Evidently, impressive *ρ*_b_, ∇^2^*ρ*_b_, and H_b_ values were noted for the XeO*_n_* ⋯ LB complexes compared with the KrO*_n_* ⋯ LB ones. For example, ∇^2^*ρ*_b_ values of XeO_4_ ⋯ and KrO_4_ ⋯ NCH complexes were 0.0534 and 0.0328 au, along with *E*_int_ of –4.18 and –2.23 kcal mol^−1^. Notably, higher *ρ*_b_, ∇^2^*ρ*_b_, and H_b_ values were registered for the σ-hole interactions than for the lp-hole counterparts. For instance, the *ρ*_b_ of the KrO_3_ ⋯ NH_3_ complex were 0.0236 and 0.0074 au for the former and latter interactions, respectively.

### 2.5. Noncovalent interaction analysis

**Figure 7.**
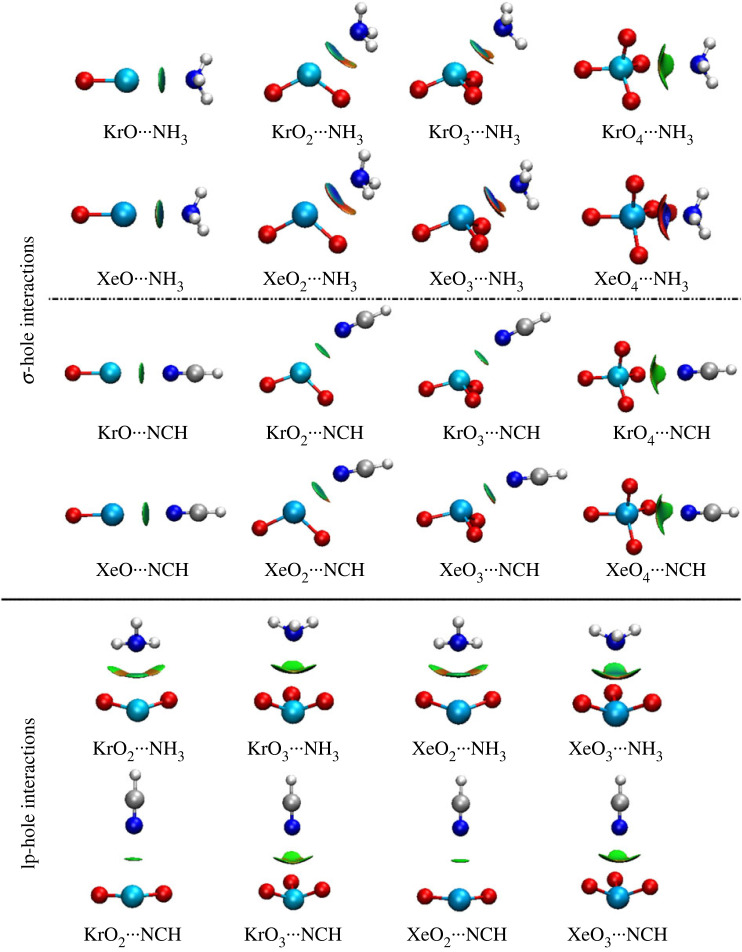
Three-dimensional NCI plots of the ZO*_n_* ⋯ LB complexes (where Z = Kr and Xe, *n* = 1, 2, 3 and 4, and LB = NH_3_ and NCH).

Electron density and its derivatives are presented as valid indexes to characterize NCI [71]. Three-dimensional NCI plots were created with a reduced density gradient value of 0.50 au. The scope of colour scale was initiated from blue (−0.035 au) to red (0.020 au). Three-dimensional NCI plots of the ZO*_n_* ⋯ LB complexes (where Z = Kr and Xe, *n* = 1, 2, 3, and 4, and LB = NH_3_ and NCH) are illustrated in figure 7.

Green-bluish-coded regions in figure 7 were generally noted between the σ-hole and N atom, revealing the strong σ-hole interactions exhibited by the studied aerogen oxides. In particular, for the XeO_2_ ⋯ , XeO_3_ ⋯ , and XeO_4_ ⋯ NH_3_ complexes, a clear blue-coded region (i.e. maximal attractive forces) was perceived, evincing their partial covalent nature that coincides with the claims of QTAIM topological parameters. On the other hand, for lp-hole interactions, green-coded regions were observed, unveiling the contributions of the coplanar oxygen substituents.

### 2.6. Symmetry-adapted perturbation theory calculations

**Figure 8.**
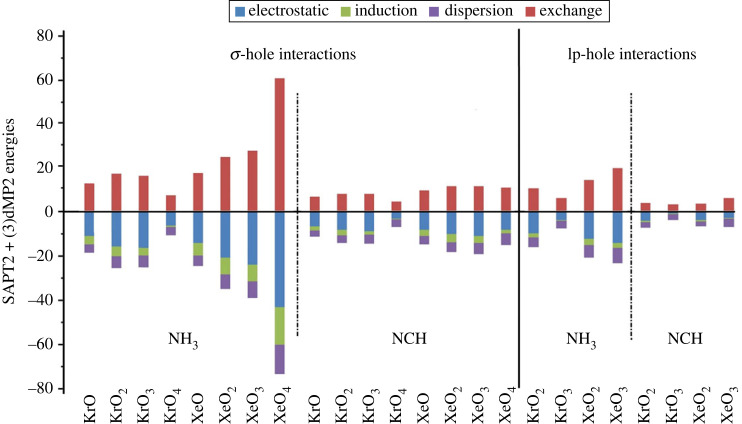
Bar chart illustrating the physical candidates of the ZO*_n_* ⋯ LB complexes (where Z = Kr and Xe, *n* = 1, 2, 3 and 4, and LB = NH_3_ and NCH).

The calculations of SAPT were dedicated to uncovering the physical forces beyond the occurrence of intermolecular interactions [72]. Figure 8 demonstrates the overall attractive components versus the repulsive candidates of the ZO*_n_* ⋯ LB complexes (where Z = Kr and Xe, *n* = 1, 2, 3, and 4, and LB = NH_3_ and NCH).

The bar chart presented in figure 8 shows *E*_elst_ energies with negative values for almost all ZO*_n_* ⋯ LB complexes. Besides, remarkable contributions were recorded for the *E*_disp_ and *E*_ind_ energies. These observations indicate the attractive nature of such forces towards stabilizing all ZO*_n_* ⋯ LB complexes, which are in line with their halogen-based interactions that are dominated by electrostatic and dispersion forces [33].

On the other hand, a repulsive nature was discerned for the exchange forces with positive *E*_exch_ values. Although the exchange forces showed significant repulsive roles, the total attractive ones were recorded with higher contributions. For instance, *E*_elst_, *E*_ind_, *E*_disp_, and *E*_exch_ values of KrO ⋯ NH_3_ complex were −11.07, −4.03, −3.66, and 12.79 kcal mol^−1^, respectively.

SAPT calculations were performed, as an example, for the XeO_3_ … NH_3_ complex within a 2–4 Å distance range with a 0.5 Å step size (electronic supplementary material, figure S2). Electronic supplementary material, figure S2, shows a direct relationship between all the physical candidates and the Xe ⋯ N distance. The *E*_elst_ forces were also found to escalate with increasing Xe ⋯ N distances, which was in line with PoC observations, as shown in figure 3.

**Figure 9.**
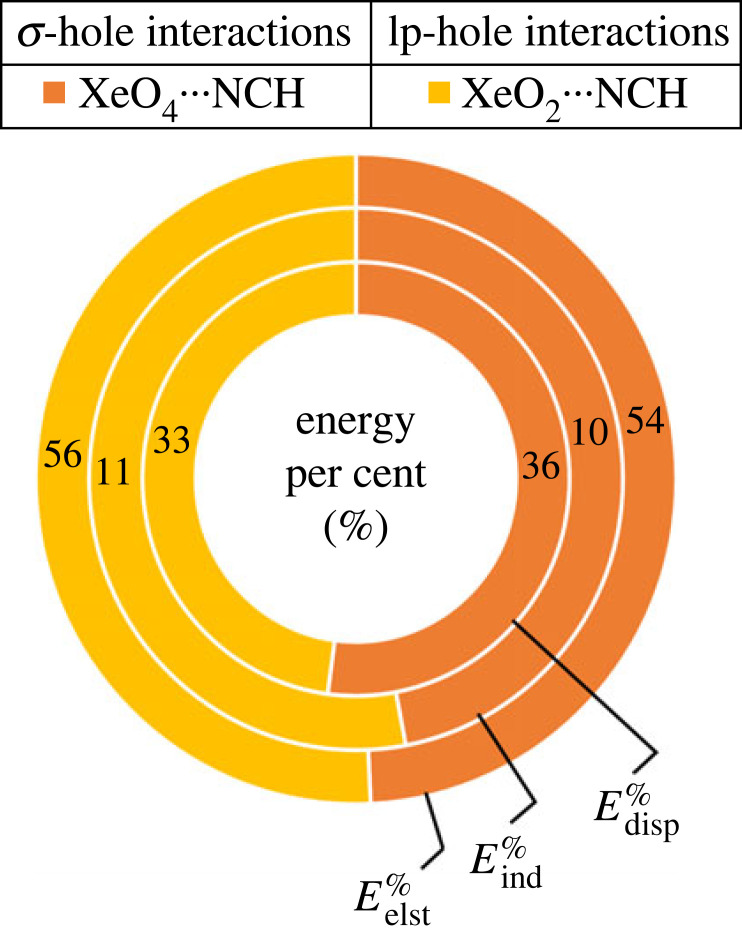
Doughnut plot illustrating the energy percent (*E*^%^) of each attractive component (i.e. $E_{\mathrm{elst}}^{\%}$*,* $E_{\mathrm{ind}}^{\%}$*,* and $E_{\mathrm{disp}}^{\%}$) to the total attractive forces of the σ- and lp-hole interactions within the XeO_4_ ⋯ and XeO_2_ ⋯ NCH complexes, respectively.

The energy percent (*E*^%^) calculations were invoked to demonstrate the contributions of the attractive components, namely $E_{\mathrm{elst}}^{\%}$, $E_{\mathrm{ind}}^{\%}$, and $E_{\mathrm{disp}}^{\%}$, to the total attractive energy within the ZO_*n*_ ⋯ LB complexes, and graphed in a doughnut plot (electronic supplementary material, figure S3). According to the illustrated data in electronic supplementary material, figure S3, the predomination of electrostatic forces was observed for all the ZO*_n_* ⋯ LB complexes, where the $E_{\mathrm{elst}}^{\%}$ contributed around 50–65% of the total attractive forces. For instance, $E_{\mathrm{elst}}^{\%}$ contributions to the attractive forces of the σ-hole and lp-hole interactions within the XeO_4_ ⋯ and XeO_2_ ⋯ NCH complexes were 54% and 56%, respectively, as shown in figure 9.

In general, the overall contributions of the attractive forces within the ZO*_n_* ⋯ LB complexes were shown to follow the pattern of electrostatic > dispersion > induction forces. For example, in the case of the XeO_3_ ⋯ NH_3_ complex, the $E_{\mathrm{elst}}^{\%}$, $E_{\mathrm{disp}}^{\%}$, and $E_{\mathrm{ind}}^{\%}$ were 64%, 21%, and 15%, respectively.

## 3. Computational methods

Chemical systems were investigated using the MP2 method, employing the aug-cc-pVTZ basis set for all atoms and the pseudopotentials (PP) for the Kr/Xe atoms. Quantum mechanical computations were performed using Gaussian 09 software [73]. As an initial step, geometry optimization was executed for the studied monomers and complexes. Upon the optimized aerogen-bearing (ZO*_n_*) molecules, electrostatic potential (EP) analysis was performed using 0.002 au electron density envelope to provide an accurate description of the EP within the chemical systems [74,75]. Molecular electrostatic potential (MEP) maps were created followed by the maximum electrostatic potential surface (*V*_s,max_) values. *V*_s,max_ values were extracted with the aid of the Multiwfn 3.7 software [76].

The electrostatic viewpoints of σ-hole [77,78] and lp-hole [13] interactions of the considered aerogen oxides were produced via point-of-charge (PoC) calculations (figure 1*a*). Variant values of PoC = –0.25 and –0.75 au were employed to demonstrate the impacts of Lewis basicity on the targeted interactions by comparing the molecular stabilization energy (*E*_stabilization_) [15]. The assessment of *E*_stabilization_ was invoked at σ-hole ⋯ /lp-hole ⋯ PoC distances in the range of 2.5–5.0 Å with a 0.1 Å step size (equation (3.1)):

3.1 $$E_{\mathrm{stabilization}}=E_{\mathrm{Hole}\text{-}\mathrm{containing}\;\mathrm{molecule}\cdot \cdot \cdot \mathrm{PoC}}-E_{\mathrm{Hole}\text{-}\mathrm{containing}\;\mathrm{molecule}}.$$

The ZO*_n_* ⋯ LB complexes (where Z = Kr and Xe, *n* = 1, 2, 3 and 4, and LB = NH_3_ and NCH) were first optimized using the MP2/aug-cc-pVTZ(PP) level of theory (figure 1*b*). No vibrational frequency calculations were performed for the investigated complexes; thus, the structures might not be energetic minima. Interaction energies (*E*_int_) were quantified and corrected from the basis set superposition error (BSSE) using the counterpoise correction (CP) method [79]. The computed *E*_int_ values were subsequently benchmarked via the CCSD(T)/CBS level of theory through equations (3.2)–(3.4). Detailedly, the MP2/CBS energy was computed using the two-point extrapolation method [80,81], where the correlation energy for the aug-cc-pVXZ basis set is proportional to X^–3^. The MP2/CBS limit energy (*E*_MP2/CBS_) values were calculated by extrapolating the *E*_MP2/aug-cc-pVQZ_ and *E*_MP2/aug-cc-pVTZ_ energy values, as demonstrated in equation (3.3).

3.2 $$E_{\mathrm{CCSD}(T)/\mathrm{CBS}}=\Delta E_{\mathrm{MP}2/\mathrm{CBS}}+\Delta E_{\mathrm{CCSD}(T)},$$

3.3 $$\Delta E_{\mathrm{MP}2/\mathrm{CBS}}=(64E_{\mathrm{MP}2/\mathrm{aug}\text{-}\mathrm{cc}\text{-}\mathrm{pVQZ}}-27E_{\mathrm{MP}2/\mathrm{aug}\text{-}\mathrm{cc}\text{-}\mathrm{pVTZ}})/37$$

3.4 $$\mathrm{and}\;\Delta E_{\mathrm{CCSD}(T)}=E_{\mathrm{CCSD}(T)/\mathrm{aug}\text{-}\mathrm{cc}\text{-}\mathrm{pVDZ}}-E_{\mathrm{MP}2/\mathrm{aug}\text{-}\mathrm{cc}\text{-}\mathrm{pVDZ}}.$$

The fundamental forces of the considered interactions were pinpointed with the execution of the symmetry-adapted perturbation theory (SAPT) analysis. With the aid of the PSI4 code, SAPT analysis was performed for the ZO*_n_* ⋯ LB complexes at the SAPT2 + (3)dMP2 level of truncation using the aug-cc-pVTZ basis set [82–84]. The fundamental forces, comprising electrostatic (*E*_elst_), induction (*E*_ind_), dispersion (*E*_disp_) and exchange (*E*_exch_) energies, were computed from equations (3.5)–(3.9) [85]:

3.5 $$E_{\mathrm{int}}^{\mathrm{SAPT}2+(3)\mathrm{dMP}2}=E_{\mathrm{elst}}+E_{\mathrm{ind}}+E_{\mathrm{disp}}+E_{\mathrm{exch}},$$

where

3.6 $$E_{\mathrm{elst}}=\;E_{\mathrm{elst}}^{\left(10\right)}+E_{\mathrm{elst}}^{\left(12\right)}+E_{\mathrm{elst}}^{\left(13\right)},$$

3.7 $$E_{\mathrm{ind}}=\;E_{\mathrm{ind},\mathrm{resp}}^{\left(20\right)}+E_{\mathrm{exch}-\mathrm{ind},\mathrm{resp}}^{\left(20\right)}+E_{\mathrm{ind},\mathrm{resp}}^{\left(22\right)}+E_{\mathrm{exch}-\mathrm{ind},\mathrm{resp}}^{\left(22\right)}+\;\delta E_{HF}^{\left(2\right)}+\delta E_{MP2\;}^{\left(2\right)},$$

3.8 $$E_{\mathrm{disp}}=\;E_{\mathrm{disp}}^{\left(20\right)}+E_{\mathrm{exch}-\mathrm{disp}}^{\left(20\right)}+E_{\mathrm{disp}}^{\left(21\right)}+E_{\mathrm{disp}\;}^{\left(22\right)}(SDQ)+E_{\mathrm{disp}}^{\left(22\right)}T+E_{\mathrm{disp}}^{\left(30\right)}$$

3.9 $$\mathrm{and}\;E_{\mathrm{exch}}=\;E_{\mathrm{exch}}^{\left(10\right)}+E_{\mathrm{exch}}^{\left(11\right)}+E_{\mathrm{exch}}^{\left(12\right)}.$$

The calculations of attractive force percent ($E_{\mathrm{attractive}}^{\%}$) were performed to manifest the contribution of each attractive component (*E*_attractive component_)—namely electrostatic ($E_{\mathrm{elst}}^{\%}$), induction ($E_{\mathrm{ind}}^{\%}$), and dispersion ($E_{\mathrm{disp}}^{\%}$)—to the total attractive forces (*E*_total attractive forces_) via equation (3.10):

3.10 $$E_{\mathrm{attractive}\;}^{\text{\%}}=\left(\frac{E_{\mathrm{attractive}\;\mathrm{component}}}{E_{\mathrm{total}\;\mathrm{attractive}\;\mathrm{force}}}\right)\times 100.$$

The nature of investigated hole interactions was identified via the analyses of the quantum theory of atom in molecule (QTAIM) protocol [70] and noncovalent interactions (NCI) index [86] using Multiwfn 3.7 software [76]. Visual Molecular Dynamics (VMD) 1.9.2 software was used to build the QTAIM schemes and NCI plots [87].

## 4. Conclusion

A comparative investigation of the potentiality of aerogen oxides (ZO*_n_*, where Z = Kr and Xe, and *n* = 1, 2, 3, and 4) to participate in hole interactions, based on their bonding sites, was established. MEP maps manifested the occurrence of σ-hole over the molecular entities of all the studied aerogen oxides. For ZO_2_ and ZO_3_ molecules, an analogous region that is oppositely located to the lone-pair (i.e. lp-hole) was evident. *E*_int_ manifestations showed that σ-hole interactions became more preferable as Z atom enlarged (i.e. Kr < Xe) according to the following pattern: KrO_4_ ⋯ < KrO ⋯ < KrO_3_ ⋯ < KrO_2_ ⋯ LB and XeO_4_ ⋯ < XeO ⋯ < XeO_2_ ⋯ < XeO_3_ ⋯ LB complexes. An exception for XeO*_n_* ⋯ NH_3_ complexes was noted for the XeO_4_⋯ NH_3_ complex, which proclaimed the most paramount *E*_int_. This exception could be ascribed to the dominance of attractive *σ*-hole ⋯ NH_3_ forces over the repulsive ones of the coplanar oxygen atoms with NH_3_. For lp-hole interactions, significant *E*_int_ were obtained for ZO_2_ ⋯ LB complexes over ZO_3_ ⋯ LB candidates, which were in line with *V*_s,max_ calculations. QTAIM and NCI analyses suggested the occurrence of the examined hole interactions and highlighted the partial covalent nature of XeO_2_ ⋯ , XeO_3_ ⋯ , and XeO_4_ ⋯ NH_3_ complexes. SAPT calculations revealed significant contributions of the electrostatic ($E_{\mathrm{elst}}^{\%}$) force, which represented about 50–65% of the total attractive forces within almost all the ZO*_n_* ⋯ LB complexes. These findings will be relevant for forthcoming research on aerogen oxide-based complexes and numerous applications, including in supramolecular chemistry and crystal engineering.

## Data Availability

All relevant necessary data to reproduce all results in the paper are within the main text and electronic supplementary material. Supplementary material is available online [88].
